# Presynaptic chloride-dependent regulation of spontaneous glutamate release in the rat medial preoptic nucleus

**DOI:** 10.3389/fncel.2026.1800250

**Published:** 2026-04-09

**Authors:** Mario Pérez-del-Pozo, Tatiana Kuznetsova, Staffan Johansson, Michael Druzin

**Affiliations:** Department of Medical and Translational Biology, Umeå University, Umeå, Sweden

**Keywords:** allopregnanolone, chloride, GABA_A_-receptor, glutamate, glycine receptor, hypothalamus, KCC2, presynaptic

## Abstract

Neurosteroids and inhibitory neurotransmitters can modulate neurotransmitter release from presynaptic terminals, yet the mechanisms underlying such modulation remain unclear. In this study, we investigated how presynaptic glycine and GABA_A_R-receptors (GlyRs and GABA_A_Rs) regulate glutamate release onto neurons in the medial preoptic nucleus (MPN), a hypothalamic region critically involved in reproductive and social behaviors. Using patch-clamp recordings from mechanically dissociated MPN neurons with functionally preserved presynaptic terminals, we selectively examined local presynaptic effects of receptor activation. Both the neurosteroid allopregnanolone and the selective GABA_A_R agonist muscimol consistently increased the frequency of glutamate-mediated spontaneous excitatory postsynaptic currents (sEPSCs). This facilitation was sensitive to the GABA_A_R-blocker picrotoxin, abolished by inhibition of sodium-potassium-chloride cotransporter 1 (NKCC1) or by the sodium-channel blocker tetrodotoxin, consistent with a mechanism involving depolarizing chloride efflux driven by a high intraterminal chloride ion concentration and subsequent sodium-dependent recruitment of presynaptic calcium channels. In contrast, activation of presynaptic GlyRs produced bidirectional effects on glutamate release: facilitation in some terminals and inhibition in others. We demonstrate that the inhibitory effect likely depends on low intraterminal chloride concentration maintained by the potassium-chloride cotransporter 2 (KCC2), which enables chloride influx and hyperpolarization upon GlyR activation. Consistent with this mechanism, pharmacological blockade of chloride extrusion abolished glycine-induced inhibition, and immunogold labeling revealed KCC2 presence in a subset of presynaptic terminals innervating MPN neurons. Together, these findings suggest functional presynaptic KCC2 in central neurons and identify presynaptic chloride homeostasis as a key determinant of synapse-specific modulation of glutamate release in the MPN.

## 1 Introduction

Chloride-permeable GABA type A receptors (GABA_A_Rs) and glycine receptors (GlyRs) are ligand-gated ion channels that mediate most fast synaptic inhibition in the central nervous system (CNS). The inhibitory effect of these receptors depends on the chloride gradient across the neuronal membrane, which normally favors chloride influx (Legendre, 2001; Goetz et al., 2007; Dutertre et al., 2012). This influx produces postsynaptic hyperpolarization and reduces the probability of action potential generation. The chloride gradient in neurons is primarily established by two cation–chloride cotransporters: the sodium-potassium-chloride cotransporter 1 (NKCC1), which promotes chloride uptake, and the neuron-specific potassium-chloride cotransporter 2 (KCC2), which is responsible for chloride extrusion (Blaesse et al., 2009). In mammals, KCC2 expression undergoes developmental upregulation, shifting GABAergic and glycinergic signaling from an initially depolarizing and excitatory mode to a predominantly inhibitory mode. This transition reflects the progressive reduction of intracellular chloride concentration as KCC2 activity increases (Rivera et al., 1999; Ben-Ari et al., 2007; Takayama and Inoue, 2010). The onset and level of KCC2 expression vary across neuronal populations and developmental stages, thereby contributing to heterogeneity in the maturation of inhibitory synaptic transmission and circuit formation (Zhang et al., 2006; Szrinivasan et al., 2025). KCC2 remains tightly regulated throughout postnatal development and adulthood through post-translational modifications and protein–protein interactions controlling its trafficking and membrane stability (Lee H. H. C. et al., 2011; Lee M. et al., 2011; Kahle et al., 2013). Disruption of this regulation impairs neuronal function and contributes to disorders such as epilepsy (Palma et al., 2006; Kahle et al., 2016), neuropathic pain (Coull et al., 2003; Jolivalt et al., 2008; Kahle et al., 2014; Zhao S. et al., 2022), and neurodevelopmental diseases (Pisella et al., 2019).

In healthy adult neurons, KCC2 activity in somatic and dendritic compartments maintains a low intracellular chloride concentration, thus ensuring that activation of GABA_A_Rs and GlyRs produces effective inhibition. However, accumulating evidence indicates that activation of GABA_A_Rs and GlyRs at presynaptic terminals may not inhibit but instead can facilitate neurotransmitter release. Presynaptic GABA_A_Rs have been shown to promote long-term potentiation of synapses (Ruiz et al., 2010) and to induce spontaneous GABA release (Haage et al., 2002; Xiao et al., 2007). Other studies have demonstrated that presynaptic GABA_A_Rs enhance glutamate release (Stell et al., 2007; Kim et al., 2011; Iwata et al., 2013). Similarly, presynaptic GlyRs facilitate both glutamatergic (Turecek and Trussell, 2001; Lee et al., 2009; Kunz et al., 2012) and GABAergic transmission (Choi et al., 2013). Taken together, these findings indicate that activation of presynaptic ligand-gated chloride channels can depolarize presynaptic terminals, consistent with elevated intracellular chloride concentrations in the presynaptic compartment. This, in turn, suggests that KCC2 expression is low or even absent in presynaptic terminals, leading to a higher intracellular chloride concentration than typically found in adult neuronal cell bodies.

Yet, several studies have also reported inhibitory effects of presynaptic GABA_A_R and GlyR activation on neurotransmitter release (Duebel et al., 2006; Huang et al., 2024), along with evidence for presynaptic expression of KCC2 (Vu et al., 2000). While these effects may result from mechanisms still influenced by elevated intracellular chloride, including shunting inhibition or inactivation of voltage-gated sodium and calcium channels caused by depolarizing chloride currents, they may also reflect active KCC2-mediated chloride extrusion in certain presynaptic terminals, although its presence and functional significance in presynaptic compartment remain to be fully elucidated.

In this study, we investigated the presynaptic mechanisms underlying chloride-dependent regulation of spontaneous glutamate release onto neurons in the rat medial preoptic nucleus (MPN). Using patch-clamp recordings of spontaneous glutamatergic excitatory postsynaptic currents (sEPSCs), we demonstrate that activation of presynaptic GABA_A_Rs predominantly enhances glutamate release, whereas activation of presynaptic GlyRs produces either potentiation of release or, in a substantial fraction of terminals, a hyperpolarization-dependent reduction of release. Importantly, pharmacological blockade of chloride extrusion abolished this inhibitory effect. Together with immunogold electron microscopy, our results suggest that KCC2 may be present in some presynaptic terminals and are compatible with KCC2-mediated chloride extrusion in a subset of GlyR-expressing glutamatergic terminals within the MPN, where it appears to determine the effect of presynaptic GlyR activation on spontaneous glutamate release.

## 2 Materials and methods

### 2.1 Animals and cell preparation

Male Sprague Dawley rats (21–35 days old) were housed in a controlled environment at 21 °C under a 12 h day/night cycle. Animals were weaned at 21 days of age and kept at no more than four per cage with *ad libitum* access to water and food.

Brains were obtained by decapitation without the use of anesthetics, rapidly extracted, and transferred to pre-oxygenated, ice-cold cutting solution (see below). Coronal slices (300 μm thick) containing the medial preoptic area were prepared using a 752 M Vibroslice (Campden Instruments Limited). Slices were incubated for at least 30 min at 32 °C in oxygenated recovery solution (see below). For all electrophysiological experiments, acutely dissociated neurons from the medial preoptic nucleus were obtained by mechanical dissociation without enzymes.

### 2.2 Electrophysiological recordings

Whole-cell membrane currents were recorded with the amphotericin B-perforated patch method, as previously described (Haage et al., 2002; Yelhekar et al., 2017) using an Axopatch 700A amplifier, a Digidata 1322A interface, and the pCLAMP software (version 9.2; all from Axon Instruments). Patch pipettes with 3–4 MΩ tip resistance were pulled from borosilicate capillaries (Harvard Apparatus, United Kingdom) using a micropipette puller (Model P-97, Sutter Instrument CO, United States) and backfilled with intracellular solution (see below). The liquid-junction potential (12 mV), was calculated according to the stationary Nernst–Planck equation using LJPcalc (RRID: SCR_025044) (Marino et al., 2014) and was compensated in the voltages given. Test and control solutions were applied via a gravity-driven perfusion system with the outlet of an eight-barrel pipette positioned 100–200 μm from the recorded cell.

### 2.3 Solutions and drugs

To facilitate the interpretation of results, only HEPES-based solutions were used in all experimental procedures to exclude bicarbonate and thereby prevent confounding anion fluxes through GABA_A_Rs and GlyRs.

The cutting solution contained (in mM): 87 NaCl, 2.5 KCl, 0.5 CaCl_2_, 7.0MgCl_2_, 10 HEPES, 70 sucrose, 25 D-glucose. The recovery solution contained (in mM): 124 NaCl, 3.0 KCl, 2.0 CaCl_2_, 6.0 MgCl_2_, 2.0 MgSO_4_, 10 HEPES, 20 sucrose, 10 D-glucose. The standard extracellular solution contained (in mM): 116.6 NaCl, 23.4 NaAcetate, 3 KCl, 1.5 CaCl_2_, 1.2 MgCl_2_, 10 HEPES, 10 D-glucose. For all extracellular solutions, pH was adjusted to 7.4 using NaOH. The intracellular solution contained (in mM): 140 CsAc, 3.0 NaAc, 1.2 MgCl_2_, 1.0 EGTA, 10 HEPES, 10 sucrose with pH adjusted to 7.2 with CsOH and supplemented with 400 μM amphotericin B (Sigma-Aldrich) dissolved in DMSO. Osmolarity of all solutions was adjusted to 300 mOsm with sucrose. Standard extracellular solution was used as control, and test solutions were prepared by supplementing it with one or more of the following compounds: picrotoxin (Sigma-Aldrich), bumetanide (Sigma-Aldrich), allopregnanolone (Sjukhusapoteket Umeå), glycine (Fisher Scientific), muscimol (Tocris Bioscience), VU0463271 (Tocris Bioscience), or tetrodotoxin (Latoxan).

### 2.4 Data analysis

Individual postsynaptic currents were detected semi-automatically using MiniAnalysis software (Synaptosoft Inc.).

To estimate average (population-level) treatment effects while accounting for nesting within animals, a linear mixed-effects model was fit to bin-level frequencies with animal included as a random intercept:


$$\log\;(1+{\mathrm{Hz}}_{\mathrm{ij}})=\beta_{0}+\beta_{1}\;{\text{condition}}_{\mathrm{ij}}+u_{0j}+\varepsilon_{\mathrm{ij}},$$

where i indexes bins and j animals; ${\text{condition}}_{\mathrm{ij}}$ is 0 for baseline and 1 for drug, $u_{0j}∼N(0,\sigma_{u}^{2})$ , and $\varepsilon_{\mathrm{ij}}∼N(0,\sigma^{2})$ . The log(Hz + 1) link was used to stabilize variance and keep zeros defined; the fixed effect $\beta_{1}$ is reported on the log scale and, where needed, back-transformed to an approximate percent change via $\%\Delta \approx 100\cdot (\exp(\beta_{1})-1)$ with its 95% CI (Wald), and the p-value.

For within-cell paired comparisons the Wilcoxon signed-rank test on paired per-cell means was used and reported as the Wilcoxon statistic W and the exact p-value.

Effect sizes were evaluated using:

Wilcoxon r (Rosenthal’s r ).


$$r=\frac{|Z|}{\sqrt{N}},$$

where Z is the normal approximation of the Wilcoxon statistic and 𝑁 is the number of pairs. Effect sizes were interpreted as small (*𝑟*≈0.1), medium (*𝑟*≈0.3), and large (*𝑟*≳0.5). This metric is reported for all Wilcoxon results.

Paired-samples Cohen’s $d_{z}$

For paired differences $d=x_{\text{drug}}-x_{\text{baseline}}$ ,


$$d_{z}=\frac{\bar{d}}{s_{d}},$$

with $s_{d}$ the SD of the paired differences. $d_{z}\;\text{was used}\;$ for direct comparisons of effect magnitudes and provided bootstrap CIs for $d_{z}\;$ when requested. Unless noted, 95% CIs for the mean were estimated via non-parametric bootstrap (*B* = 3,000–5,000 resamples; percentile method). *B* = 5,000 was used for larger cell sets and *B* = 3,000 when working with smaller subsets.

To determine whether a cell’s response to drug application exceeded its intrinsic baseline variability, we performed a within-cell permutation (sham) test. For each cell, the observed change was calculated as


$$\Delta_{\mathrm{obs}}=\bar{H}z_{\text{Drug}}-\bar{H}z_{\text{Control}}$$

A cell-specific null distribution was generated by repeatedly splitting the 12 control bins into two sets of six (pseudo-drug vs. pseudo-control) without replacement and computing $\Delta_{\text{sham}}$ for each shuffle (*B* = 3,000–5,000). Two-sided empirical p-values were then calculated relative to this null distribution.

P-values were corrected for multiple testing across cells using the Benjamini–Hochberg false discovery rate (BH-FDR).

Neurons were classified according to their response to drug application using a *Z*-score method:


$$Z=\frac{x-\mu }{\sigma }$$

where 𝜇 is the mean frequency of spontaneous postsynaptic currents during the 2-min control period preceding drug application (binned in 10-s intervals), *σ* is the corresponding standard deviation, and 𝑥 is the mean frequency of spontaneous postsynaptic currents during drug application (binned in 10-s intervals).

Responses were defined as increased when *Z* > 1, decreased when *Z* < −1, and unresponsive when −1 ≤ *Z* ≤ 1. Neurons were grouped accordingly, and the non-parametric Wilcoxon signed-rank test was used to determine the statistical significance of drug effects within each group, with *p* < 0.05 considered significant. In the statistical reporting, 𝑛 denotes the number of cells and 𝑁 the number of animals.

### 2.5 Gold immunostaining and electron microscopy

Experimental animals were deeply anesthetized with isoflurane followed by an intraperitoneal injection of sodium pentobarbital (240 mg/kg, Apoteksbolaget, Sweden). Transcardial perfusion was performed using Tyrode’s solution, followed by 2% paraformaldehyde (PFA) and 2% glutaraldehyde (GA) in 0.1 M phosphate buffer (PB, pH 7.4). After the perfusion, the brain was removed and post-fixated in the same PFA-GA solution overnight. It was later preserved in a 1% PFA-PB for short-term storage.

Dissection of 1 mm^3^ tissue block containing the MPN was performed in 4% agarose and frozen at −80 °C in cryoprotective sucrose-glycine-gelatine solution, for subsequent sectioning. Ultrathin slices (60–90 μm) were obtained using a cryomicrotome (Leica Instruments). The slices were washed in PBS (37 °C for 20 min), followed by four washes in 0.1% glycine-PBS at room temperature. The slices were then incubated (room temperature for 20 min) with blocking solution, consisting of 1% gelatine from cold-water fish skin (Sigma 7,041).

The grids were incubated (room temperature for 1 h) with the primary rabbit anti-KCC2 antibody (1:50 in blocking solution, Millipore, 07–432). Afterward, the grids were washed in 1:10 blocking solution (room temperature, 2 min, 4 times) and then incubated (room temperature for 20 min) with gold-Protein A (1:50 in blocking solution, with particle size options of 10 or 20 nm). Following incubation, the grids were washed in PBS (room temperature, 2 min, 4 times) and post-fixed in 1% GA in PBS (room temperature, 5 min). Subsequently, the sections were washed in ddH_2_O (room temperature, 2 s, 8 times) and embedded in 1% methyl cellulose (room temperature, 2 times, 1 s). After contrast staining with 5% Uranyl Acetate (room temperature for 10 min, protected from light), the sections were dried and examined using a JEOL 1230 Transmission Electron Microscope. The images were post-processed using the open-source Fiji software (Schindelin et al., 2012).

## 3 Results

### 3.1 Effects of allopregnanolone on spontaneous neurotransmission in the MPN

**Figure 1:**
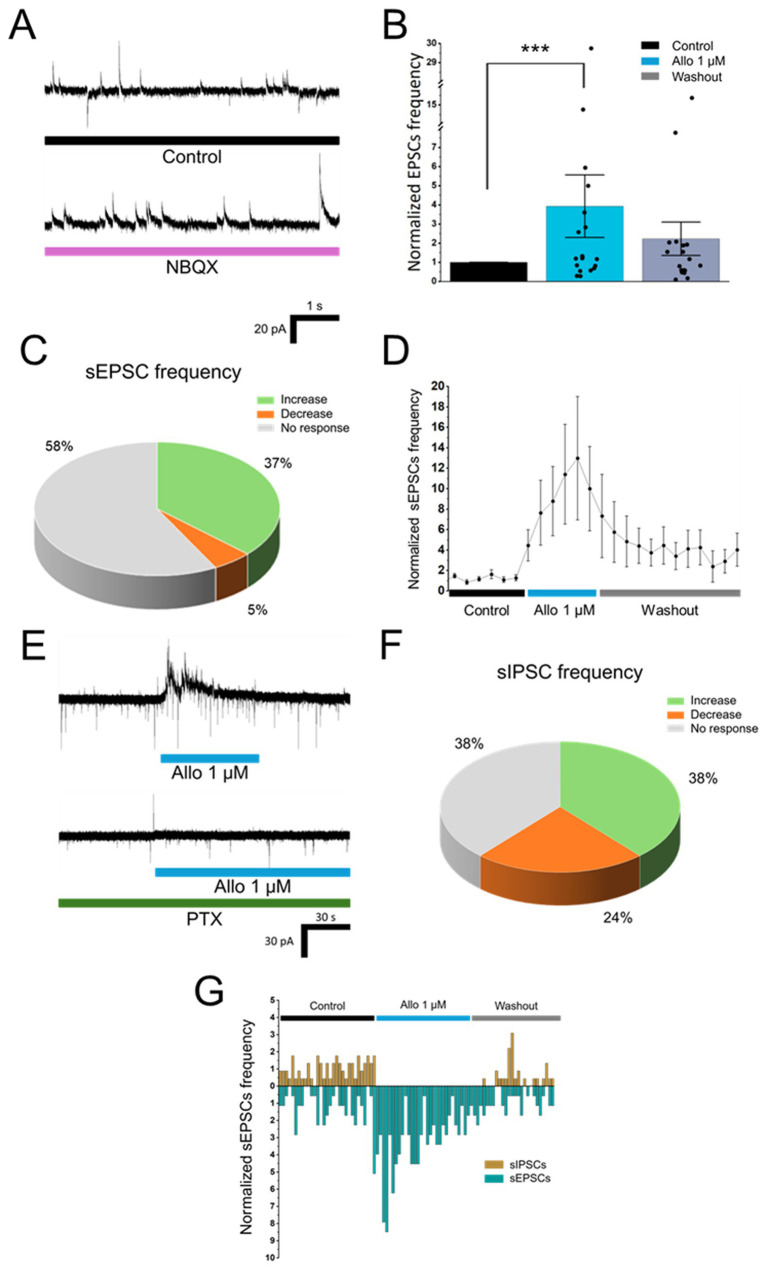
Effects of allopregnanolone on spontaneous neurotransmitter release in isolated MPN neurons. **(A)** Representative voltage-clamp recordings showing inward sEPSC and outward sIPSC under control conditions (top) and the complete abolition of sEPSC during NBQX application (bottom). **(B)** Normalized sEPSC frequency (Bin = 60 s) in response to 1.0 μM allopregnanolone application (*p* = 1.27 × 10^−14^, *n* = 19, *N* = 11). **(C)** Distribution of cells classified by *Z*-score for sEPSC frequency changes during 1.0 μM allopregnanolone (*n* = 19). **(D)** Normalized mean sEPSC frequency (Bin = 10 s) before, during 1.0 μM allopregnanolone application, and subsequent washout (*n* = 19). **(E)** Example recordings illustrating pre- (sEPSC frequency increase) and postsynaptic responses (large, long-lasting outward current) to 1.0 μM allopregnanolone in control (top) and their absence after preincubation with 100 μM picrotoxin (PTX, bottom). **(F)** Distribution of cells classified by *Z*-score for sIPSC frequency changes during 1.0 μM allopregnanolone (*n* = 13). **(G)** Example of a single cell with *Z* > 1 for sEPSC and *Z* < −1 for sIPSC, plotted in 10 s bins. Error bars indicate S. E. M.; *p < 0.05, **p < 0.01, ***p < 0.001.

Recordings of membrane currents were made from mechanically dissociated neurons from the rat medial preoptic nucleus, which retained functional adherent presynaptic terminals, using the voltage-clamp whole-cell amphotericin B perforated-patch configuration. During recordings, the holding potential was set between −52 and −62 mV (adjusted for liquid junction potential), to allow simultaneous detection of both outward spontaneous inhibitory and inward excitatory postsynaptic currents (sIPSC and sEPSC, respectively; Figure 1A), minimizing the risk of confusing these two types of events during subsequent analysis. Inward sEPSCs were detected in the vast majority of cells tested, with a mean frequency of 0.75 ± 0.12 Hz (median 0.31 Hz; *n* = 96, *N* = 76). Application of 10 μM NBQX completely abolished the inward currents (*n* = 9, *N* = 6; Figure 1A), suggesting that the sEPSCs were mediated by AMPA/kainate receptors activated by glutamate release from presynaptic terminals.

Previous studies have shown that spontaneous GABA release onto MPN neurons can be potentiated by presynaptic depolarization evoked through activation of presynaptic GABA_A_ receptors by allopregnanolone (Haage et al., 2002). Therefore, we tested whether the application of allopregnanolone modulates spontaneous glutamate release from the axon terminals that form synapses with MPN neurons.

A linear mixed-effects model analysis accounting for cells nested within animals revealed that 1.0 μM allopregnanolone significantly increased sEPSC frequency (*β* = 0.25, 95% CI 0.19 to 0.32; *p* = 1.27 × 10^−14^; *n* = 19, *N* = 11; Figure 1B). Mean sEPSC frequency increased from 0.38 ± 0.09 Hz during control to 0.97 ± 0.27 Hz during allopregnanolone application (medians: 0.22 Hz vs. 0.42 Hz). This represented a robust effect with Cohen’s *d*_z_ = 0.64 (95% CI 0.28 to 1.12), indicating a medium-to-large shift in the level of glutamate release. *Z*-score classification showed that 7 of 19 cells exhibited increased sEPSC frequency, 1 cell showed a decrease, and 11 cells were classified as unresponsive (Figure 1C).

On average, the allopregnanolone-induced increase in sEPSC frequency peaked within 30–50 s of application and gradually declined over the course of the application (Figure 1D). In most cells, the effect was reversible within 3 min, although the exact time course varied between cells. There was no significant change in the amplitude of sEPSC upon allopregnanolone application (*p* = 0.71, *n* = 19, *N* = 11), suggesting that the observed effects of allopregnanolone were presynaptic. Preincubation for 5 min in 100 μM picrotoxin, an antagonist of chloride-permeable glycine and GABA_A_ receptors, abolished the allopregnanolone-induced changes in sEPSC frequency (Figure 1E). In the presence of picrotoxin, allopregnanolone did not significantly alter sEPSC frequency, as indicated by a linear mixed-effects model with animal identity included as a random intercept (*β* = −0.04, 95% CI −0.13 to 0.05, *p* = 0.405; *n* = 8, *N* = 6).

Analysis of the group × condition interaction further demonstrated that the allopregnanolone effect was significantly attenuated under picrotoxin, reflected in a negative interaction coefficient (*β*_interaction_ = −0.29, 95% CI -0.43 to −0.15, *p* = 4.96 × 10^−5^), thus indicating the involvement of allopregnanolone-sensitive presynaptic chloride-permeable receptors in modulating glutamate release onto MPN neurons. Consistent with previous reports, the sIPSC frequency was also modulated by allopregnanolone. Although allopregnanolone increased sIPSC frequency in a subset of recorded neurons (5 out of 13 cells, based on *Z*-score analysis; Figure 1F), this enhancement did not always coincide with changes in sEPSC frequency within the same cells. Thus, some neurons showed increased sEPSC frequency without corresponding changes – or even with reductions – in sIPSC frequency (Figure 1G). These findings suggest a differential pattern of expression or functional involvement of presynaptic chloride-permeable receptors across excitatory and inhibitory terminals innervating MPN neurons.

### 3.2 Effects of presynaptic GABA_A_-receptor activation by muscimol on spontaneous glutamate release onto MPN neurons

**Figure 2:**
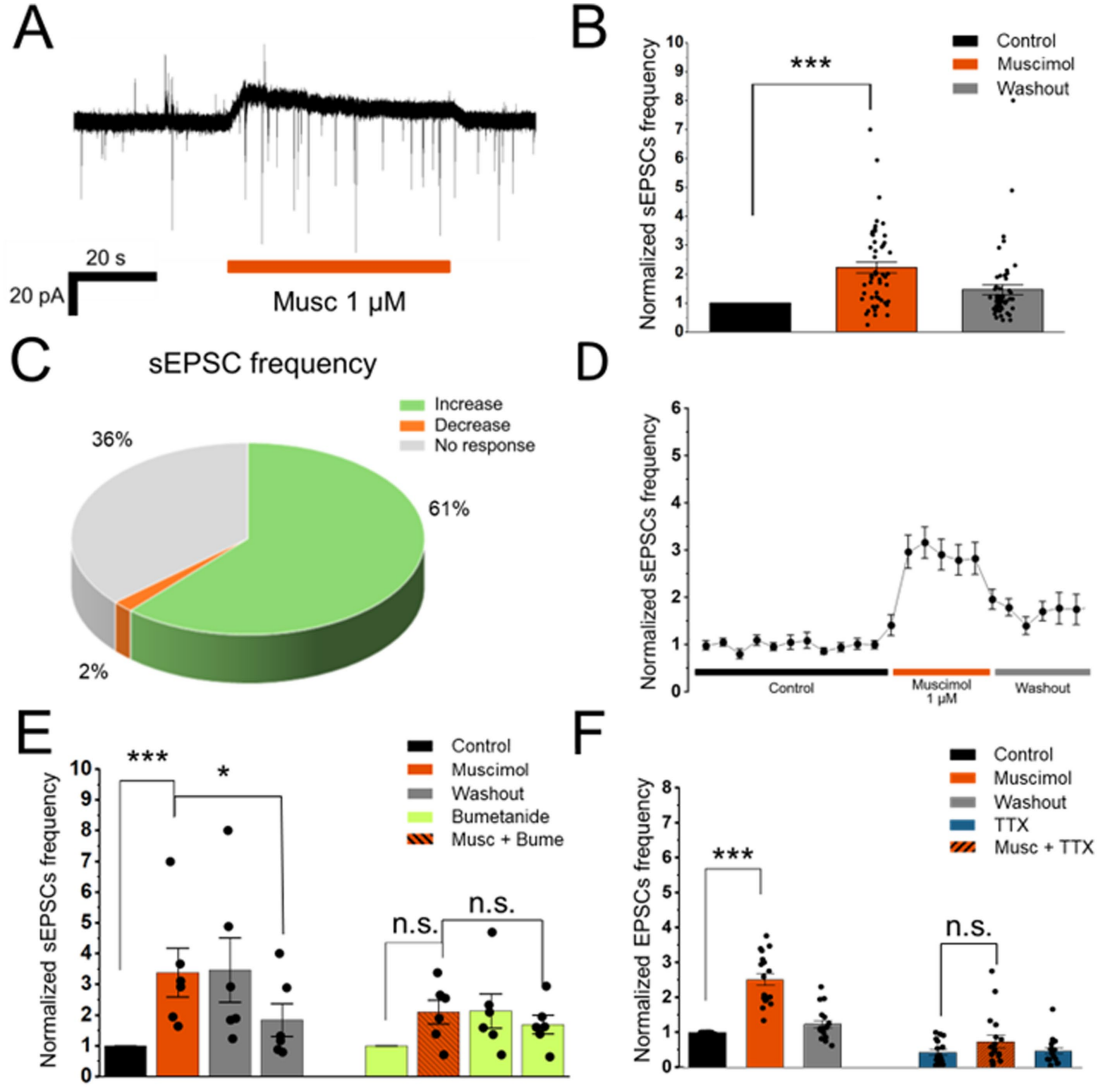
Effects of presynaptic GABA_A_R activation on spontaneous glutamate release in isolated MPN neurons. **(A)** Representative voltage-clamp recordings showing an increase in sEPSC frequency during 1.0 μM muscimol application. **(B)** Normalized sEPSC frequency (60 s bins) of cells in response to 1.0 μM muscimol (*p* = 9.12 × 10^−22^, *n* = 52, *N* = 35). **(C)** Distribution of cells classified by *Z*-score for sEPSC frequency changes during 1.0 μM muscimol application. **(D)** Normalized sEPSC frequency of increased response to muscimol cells in control, 1.0 μM muscimol, and washout conditions (Bin = 10 s; *n* = 38, *N* = 29). **(E)** Normalized sEPSC frequency during 1.0 μM muscimol application in control (*p* = 0.0312, *n* = 6) and in presence of 100 μM bumetanide (*p* = 0.44, *n* = 6, *N* = 4). Because of an unusually slow washout, an additional minute of washout is shown to demonstrate that the muscimol effect is no longer present (*p* = 0.0312). **(F)** Normalized sEPSC frequency of positively responding cells (*Z* > 1) during 1.0 μM muscimol application under control conditions and mEPSC frequency in the same cells in the presence of 2.0 μM TTX (*n* = 18, *N* = 16). Error bars indicate S. E. M.; *p < 0.05, **p < 0.01, ***p < 0.001.

Allopregnanolone is known to modulate several types of ion channels, but its primary target is believed to be GABA_A_Rs (Majewska, 1992; Lambert et al., 1995). To specifically assess the effects of presynaptic GABA_A_R activation on spontaneous glutamate release, we used muscimol, a selective and potent GABA_A_R agonist (Johnston, 2014). Similar to allopregnanolone, application of 1.0 μM muscimol produced a robust enhancement of spontaneous glutamate release (Figure 2A). A linear mixed-effects model accounting for cells nested within animals, with animal included as a random intercept, revealed a significant muscimol-induced increase in sEPSC frequency (*β* = 0.226, 95% CI 0.180 to 0.272, *p* = 9.12 × 10^−22^; *n* = 52, *N* = 35; Figure 2B), The mean sEPSC frequency increased from 1.01 ± 0.20 Hz under control conditions to 1.55 ± 0.24 Hz during muscimol application (medians 0.47 Hz vs. 1.02 Hz). The magnitude of this increase was substantially greater than that produced by allopregnanolone, as reflected by a large paired effect size (Cohen’s *d*_z_ = 0.96, 95% CI 0.64 to 1.36). At the single-cell level, *Z*-score classification further demonstrated that muscimol increased excitatory synaptic activity in the majority of cells, with 32 of 52 cells identified as positive responders, 19 as non-responsive, and only 1 cell classified as a negative responder (Figure 2C). Notably, the time course of muscimol-induced potentiation was faster than that of allopregnanolone, typically peaking within 10–20 s after drug onset and, in most cells, reversing within ~1 min of washout, indicating a rapid and reversible enhancement of glutamate release onto MPN neurons (Figure 2D).

While the absence of a significant muscimol response in some cells may reflect a lack of presynaptic GABA_A_Rs, the muscimol-induced increase in sEPSC frequency observed in most tested cells likely results from depolarization of presynaptic terminals, depending on a chloride gradient across the presynaptic membrane that favors chloride efflux. NKCC1 is known to accumulate chloride intracellularly (Haas and Forbush, 1998; Blaesse et al., 2009), thus promoting chloride efflux and depolarization upon activation of chloride-permeable GABA_A_Rs. To test whether such depolarization mediates the muscimol-induced facilitation of spontaneous glutamate release, the same positively responding cells (*Z* > 1) were recorded in control and with 3 min preincubation with 100 μM bumetanide, a selective NKCC1 inhibitor (Shankar and Brater, 2003). Under control conditions muscimol significantly increased sEPSC frequency (*W* = 0, *p* = 3.125 × 10^−2^, *n* = 6, *N* = 4; *r* = 0.90) corresponding to a per-cell mean frequency increase of 0.50 ± 0.18 Hz (Figure 2E). In contrast, when cells were recorded in bumetanide, the muscimol-induced change was smaller and not significant (*W* = 6, *p* = 0.44, *n* = 6, *N* = 4; *r* = 0.39; Figure 2E), with a mean frequency increase of 0.17 ± 0.12 Hz. A paired cell-level comparison indicated a larger facilitation of sEPSC frequency without bumetanide than with bumetanide (*W* = 3.0, *p* = 0.15; *d*_z_ = 0.734). Bumetanide alone did not significantly alter sEPSC frequency (*W* = 4, *p* = 0.88, *n* = 6, *N* = 4; *r* = 0.18). These results are consistent with a role for NKCC1-dependent Cl^−^ accumulation in supporting GABA_A_R-driven presynaptic depolarization and subsequent glutamate release.

Depolarization of the presynaptic terminal can potentiate neurotransmitter release both directly, by activating voltage-gated calcium channels and increasing calcium influx, and indirectly, by initiating or facilitating action potential generation through activation of voltage-gated sodium channels, which subsequently activates voltage-gated calcium channels (Jang et al., 2001; Yokogawa et al., 2001). To determine whether the muscimol-induced potentiation of sEPSC frequency was dependent on voltage-gated sodium channels, we used 2.0 μM tetrodotoxin (TTX), a selective and potent blocker of voltage-gated sodium channels. The effect of 1.0 μM muscimol was compared under control conditions and after TTX preincubation in the same set of positively responding cells (*Z* > 1). Application of TTX markedly reduced sEPSC frequency from 0.94 ± 0.26 Hz (median 0.51 Hz) under control conditions to 0.29 ± 0.08 Hz (median 0.19 Hz) in TTX. A paired Wilcoxon signed-rank test confirmed a highly significant reduction (*W* = 0, *p* = 2.93 × 10^−4^: *n* = 18, *N* = 14), with a very large effect size (*r* = 0.88). These results indicate that the majority of spontaneous events recorded under control conditions are voltage-gated sodium channel-dependent, while the remaining component (miniature excitatory postsynaptic currents; mEPSCs) likely relies on calcium influx through spontaneously activated voltage-gated calcium channels, as previously shown for GABA release in the MPN (Druzin et al., 2002). At the per-cell level, muscimol produced a strong facilitation of sEPSC frequency under control conditions (*W* = 0, *p* = 7.63 × 10^−6^, *n* = 18, *N* = 14; *r* = 0.89), corresponding to a mean increase of 0.93 ± 0.25 Hz (median increase 0.72 Hz). In marked contrast, when the same cells were recorded in TTX, the muscimol-induced increase was substantially smaller, not reaching statistical significance (*W* = 45, *p* = 8.14 × 10^−2^, *n* = 18, *N* = 14; *r* = 0.42) with the mean frequency increase 0.16 ± 0.07 Hz (median increase 0.06 Hz). A paired Wilcoxon signed-rank test showed that the muscimol-induced increase in sEPSC frequency was significantly larger under control conditions than in the presence of TTX (*W* = 13.0, *p* = 6.71 × 10^−4^, *r* = 0.744; *n* = 18, *N* = 14; Figure 2F), corresponding to a large paired-samples effect size (Cohen’s *d*_z_ = 0.83; *n* = 18, *N* = 14).

This indicates that presynaptic GABA_A_R-mediated depolarization was insufficient to directly recruit additional calcium channels, but sufficient to facilitate glutamate release indirectly by promoting local activation of sodium channels, which in turn activate voltage-gated calcium channels.

### 3.3 Activation of presynaptic GlyRs produces heterogeneous effects on spontaneous glutamate release onto MPN neurons

In addition to GABA_A_Rs, allopregnanolone has been shown to modulate glycine receptors (Alvarez and Pecci, 2019; Bukanova et al., 2020). To investigate whether presynaptic GlyRs contribute to the effects of allopregnanolone on glutamate release, we applied 100 μM glycine.

**Figure 3:**
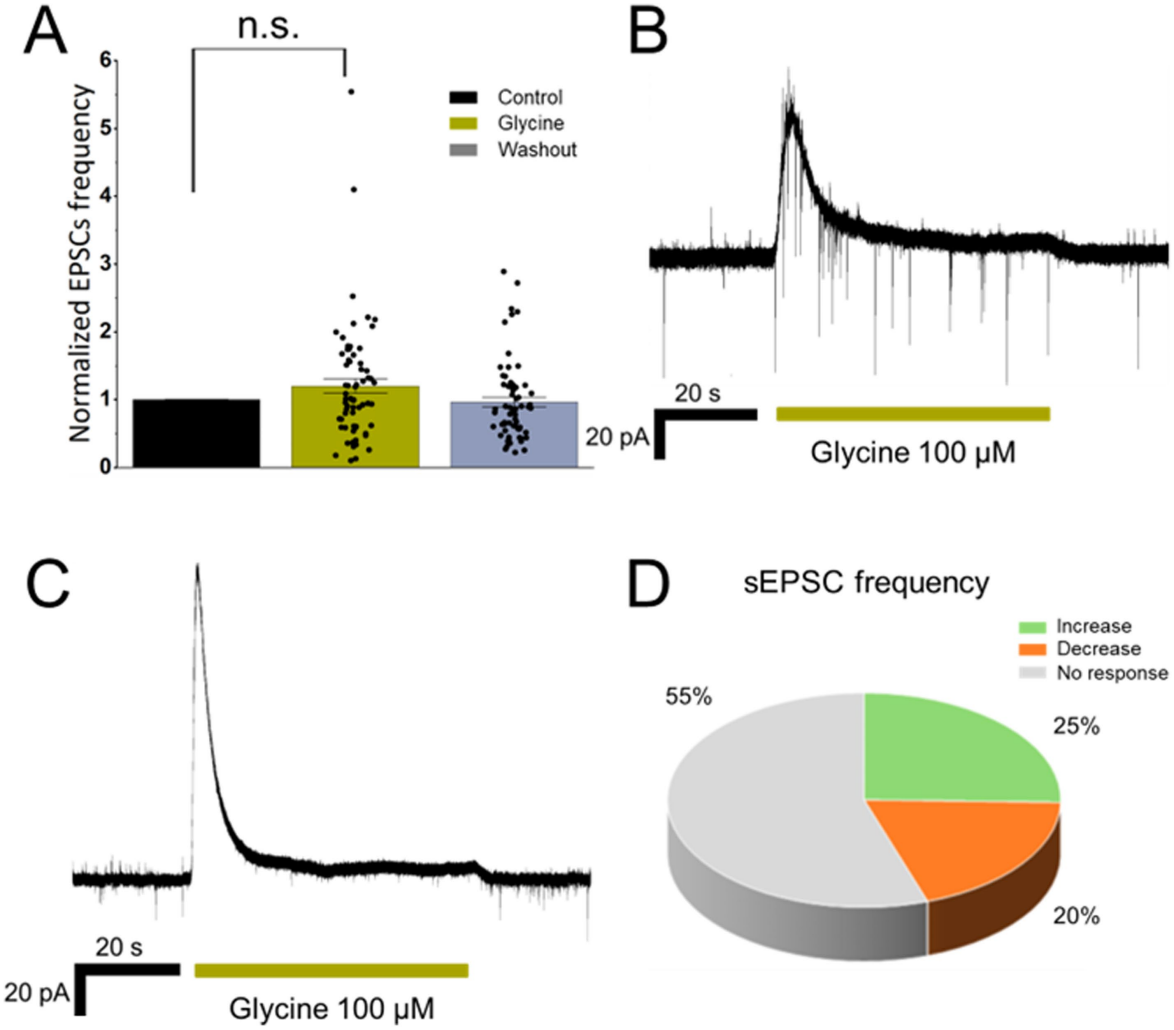
Effects of presynaptic GlyR activation on spontaneous glutamate release in isolated MPN neurons. **(A)** Normalized sEPSC frequency (Bin = 60 s) of cells in response to 100 μM glycine application (*p* = 0.44, *n* = 66, *N* = 46). Representative voltage-clamp recordings showing an increase in sEPSC frequency **(B)** and decrease **(C)** during 100 μM glycine application **(D)** Distribution of cells classified by *Z*-score for sEPSC frequency changes during 100 μM glycine application. Error bars indicate S. E. M.

A linear mixed-effects model accounting for cells nested within animals, with animal included as a random intercept, revealed no significant effect of glycine on spontaneous excitatory synaptic activity (*β* = −0.02, 95% CI −0.06 to +0.03, *p* = 0.44; *n* = 66, *N* = 46; Figure 3A). The mean per-cell sEPSC frequency under control conditions was 0.99 ± 0.14 Hz, compared to 1.00 ± 0.16 Hz during glycine (medians 0.50 Hz vs. 0.40 Hz) with a mean difference of only +0.01 Hz. This minimal population shift contrasts sharply with the diversity of single-cell responses as permutation-based cell-level analysis revealed pronounced heterogeneity in the glycine response. For each cell, a per-cell permutation test (3,000 iterations of pseudo-conditions) generated a cell-specific null distribution of the sEPSC frequency, against which the observed glycine-induced change was compared. Empirical permutation p-values, corrected for multiple testing using the Benjamini–Hochberg false discovery rate (BH-FDR), were strongly skewed toward significance (median *p*_perm_ ≈ 3 × 10^−4^), reflecting highly reliable deviations from baseline. Consistent with this analysis, *Z*-score classification based on control variability showed similarly mixed responses: among 66 cells, 20 were positive responders, 12 negative responders, and 34 non-responsive (median *Z* = 0.0098, Interquartile Range −1.38 to +0.71; Figures 3B–D). Together, these results show that glycine produces large but opposing effects at the single-cell level, resulting in no net effect in Hz across the population of recorded cells.

The time course of glycine-induced changes in sEPSC frequency was similar to that of muscimol, regardless of whether the effect was a potentiation or a reduction of frequency.

### 3.4 GlyR-dependent reduction in sEPSC frequency is consistent with hyperpolarization of the presynaptic terminals enabled by KCC2 activity

The observed reduction in sEPSC frequency upon glycine application may result from shunting inhibition or from depolarization-induced inactivation of voltage-gated sodium channels, both of which prevent the activation of calcium channels and suppress glutamate release (Ong et al., 2000; Branchereau et al., 2016). To examine these possibilities, we applied glycine in the presence of TTX, to eliminate sodium channel activity. Since TTX alone markedly reduced baseline glutamate release, potentially masking further glycine-induced suppression, we used an extracellular solution containing 8.0 mM calcium to compensate for the TTX-induced decrease, as elevated calcium has been shown to enhance action potential-independent neurotransmitter release (Xu et al., 2009). Although the baseline frequency of mEPSCs was lower under these conditions (0.60 ± 0.14 Hz, median 0.52 Hz; 95% CI 0.35 to 0.86 Hz) compared with the preceding sEPSC frequency in control (0.82 ± 0.20 Hz, median 0.64 Hz; 95% CI 0.49 to 1.24 Hz), this reduction did not reach statistical significance (Wilcoxon *W* = 3, *p* = 0.078; *n* = 7, *N* = 5). Importantly, activation of presynaptic glycine receptors in the presence TTX produced a significant reduction in mEPSC frequency to 0.25 ± 0.10 Hz (median 0.18 Hz; 95% CI 0.05 to 0.44 Hz; *W* = 0, *p* = 0.016; *n* = 7, *N* = 5) with a very large within-cell effect size (*r* = 0.89), indicating that presynaptic glycine receptor activation directly hyperpolarizes terminals rather than acting indirectly via inactivation of sodium channels.

**Figure 4:**
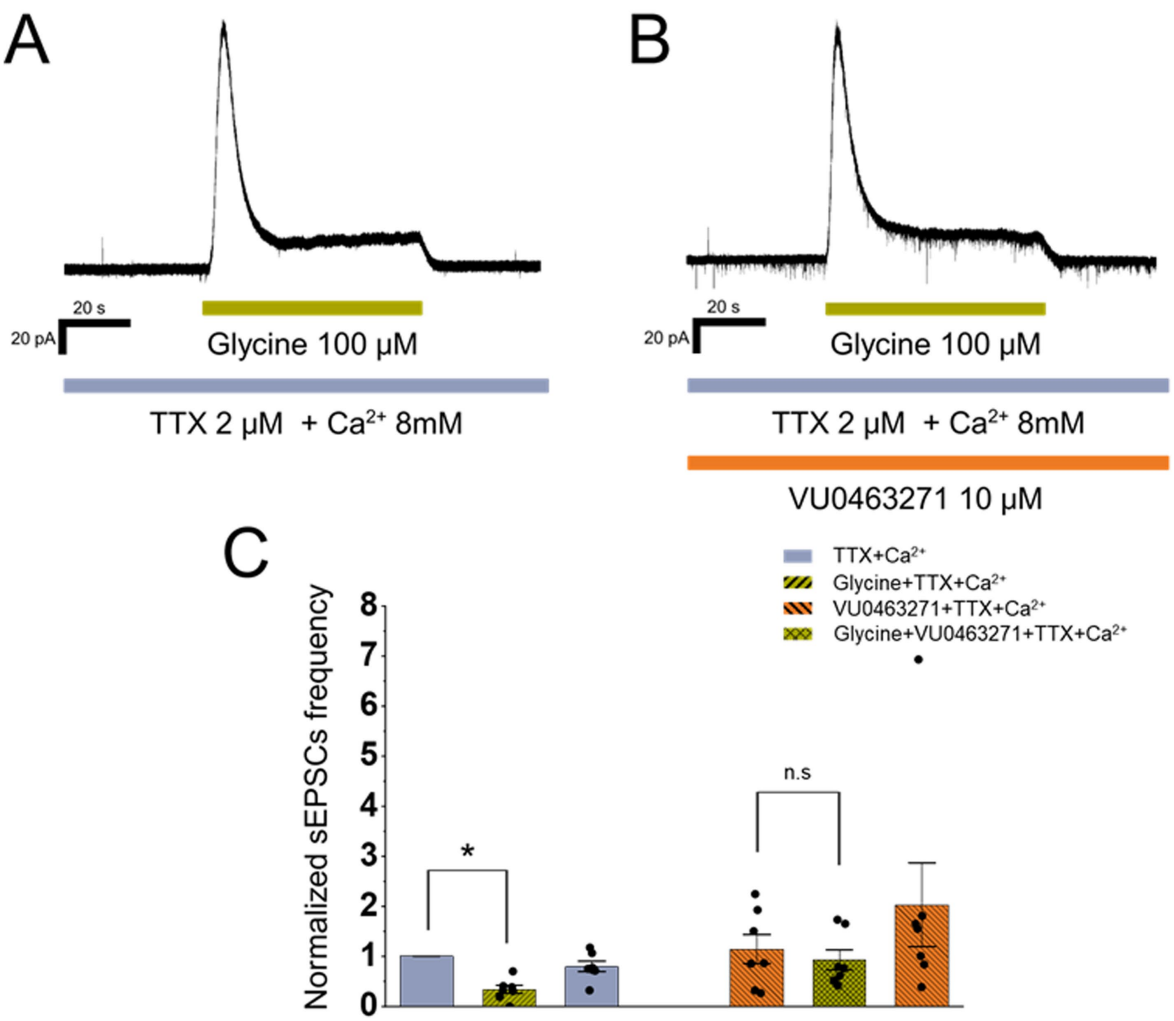
GlyR-dependent reduction of glutamate release is mediated by chloride-driven hyperpolarization. **(A)** Representative voltage-clamp recording showing the reduction of mEPSC frequency in response to 100 μM glycine application in the presence of 2.0 μM TTX and 8.0 mM Ca^2+^. **(B)** Representative voltage-clamp recording of the same cell as in **(A)** after 2 min preincubation with 10 μM VU0463271. **(C)** Normalized mEPSC frequency (Bin = 60 s) showing changes in response to 100 μM glycine application with and without preincubation with 10 μM VU0463271, recorded in the same cells. Error bars indicate S. E. M.; *p < 0.05.

For glycine receptor activation to induce hyperpolarization, the intraterminal chloride concentration must be sufficiently low to allow chloride influx upon receptor opening. Such a condition requires active chloride extrusion, which in neurons is primarily mediated by KCC2 (Blaesse et al., 2009; Chamma et al., 2012). To test this hypothesis, we examined whether the glycine-induced reduction in mEPSC frequency could be modulated by KCC2 inhibition using the potent KCC2 blocker VU0463271 (Delpire et al., 2012). Cells that exhibited a decrease in mEPSC frequency in response to glycine application (*Z* < −1) in extracellular solution containing 8.0 mM calcium and TTX (Figure 4A) were subsequently preincubated for 2 min with 10 μM VU0463271 added to the same extracellular solution before glycine was applied again (Figure 4B). Baseline mEPSC frequency was not significantly altered by VU0463271 (0.73 ± 0.22 Hz, median 0.92 Hz, 95% CI 0.35–1.15 Hz) compared with VU0463271 free conditions (0.60 ± 0.14 Hz, median 0.52 Hz, 95% CI 0.35–0.86 Hz; *W* = 10, *p* = 0.578, *n* = 7, *N* = 5, *r* = 0.26). In contrast, the marked reduction in mEPSC frequency initially produced by glycine under these recording conditions (*W* = 0, *p* = 0.016; *n* = 7, *N* = 5; *r* = 0.89) was no longer significant after KCC2 inhibition (Figure 4C). In the presence of VU0463271, glycine produced only a small decrease in mEPSC frequency (−0.26 ± 0.14 Hz), which did not reach statistical significance (Wilcoxon signed-rank test: *W* = 6, *p* = 0.219, n = 7, *N* = 5; *r* = 0.51). These results indicate that the inhibitory effect of GlyR activation on glutamate release is mediated by chloride influx-driven hyperpolarization, which likely requires functional KCC2 in presynaptic terminals.

Since both the potentiation and inhibition of glutamate release seem to depend on the chloride gradient, these findings suggest heterogeneity among glutamatergic terminals in the MPN. In a subset of 44 cells tested with both muscimol and glycine, 12 cells showed a positive response to muscimol (*Z* > 1) but no response to glycine (−1 < *Z* < 1). This pattern suggests that these cells are innervated predominantly by terminals expressing GABA_A_ receptors but lacking functional GlyRs, and that these terminals likely maintain relatively elevated intraterminal chloride levels, thereby facilitating glutamate release upon GABA_A_ receptor activation. In contrast, a second group of 5 cells exhibited inhibition of glutamate release in response to glycine (*Z* < −1) together with potentiation by muscimol (*Z* > 1). This response pattern suggests that these cells are innervated by a mixed population of presynaptic terminals, some expressing GlyRs and others expressing GABA_A_ receptors. Terminals expressing GlyRs likely maintain low intraterminal chloride levels, potentially via KCC2 activity, resulting in hyperpolarization and reduced glutamate release upon glycine receptor activation. Finally, a third group of 9 cells displayed comparable responses to both muscimol and glycine, suggesting that the presynaptic terminals contacting these cells may either co-express both receptor types or consist of mixed populations of only GlyR- or GABA_A_ receptor–expressing terminals with relatively elevated intraterminal chloride levels.

This diversity argues against a simple artifact of the mechanical dissociation procedure, which could potentially influence presynaptic chloride regulation, such perturbations would be expected to produce more uniform or unidirectional effects on transmitter release. Instead, glycine and muscimol produced receptor-dependent bidirectional modulation across cells, a pattern more consistent with intrinsic heterogeneity among presynaptic terminals.

### 3.5 Gold-immunostaining demonstrates the presence of KCC2 in presynaptic terminals

Although KCC2 has recently been identified in presynaptic terminals in the retina (Vu et al., 2000; Duebel et al., 2006; Yin et al., 2020) and spinal cord (Huang et al., 2024), its presence in central presynaptic terminals has, to our knowledge, not been previously documented. We therefore investigated the subcellular localization of KCC2 and its potential association with presynaptic terminals using the pre-embedding immunogold technique in brain sections containing the MPN.

**Figure 5:**
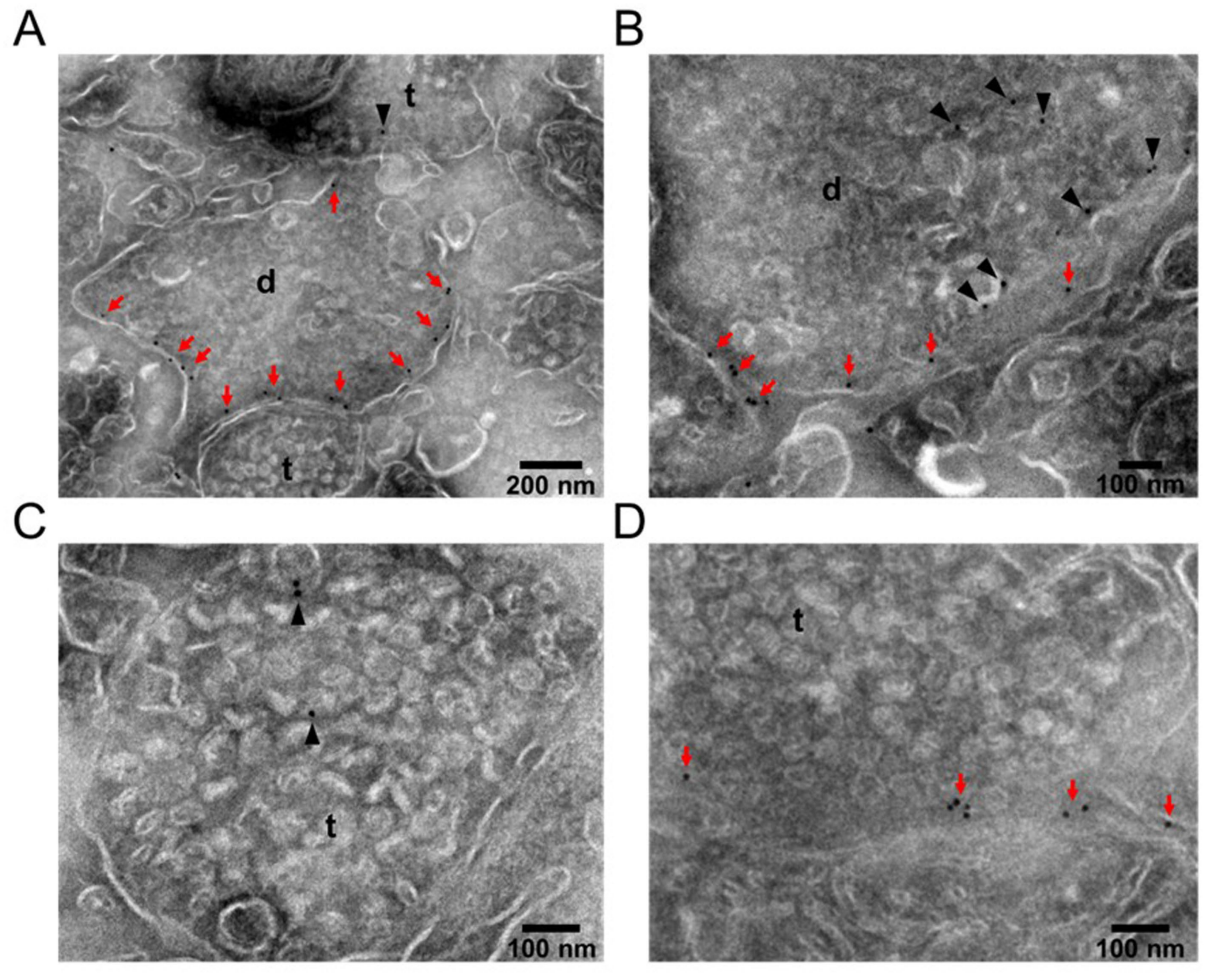
Immunogold staining of KCC2 in pre- and postsynaptic areas. **(A)** Image of a dendritic spine **(D)** with gold particles distributed across the membrane (red arrows) in close proximity of presynaptic terminals (t) containing synaptic vesicles. **(B)** Dendrite showing gold particles in the membrane (red arrows) as well as inside the cytoplasm (black arrowheads). **(C)** Presynaptic terminal with gold particles labeling KCC2 inside the terminal. **(D)** Presynaptic terminal showing KCC2 distributed across the membrane.

KCC2-associated gold particles were found to be quasi-randomly distributed, with the highest density in postsynaptic domains, identified by the absence of synaptic vesicles. Most of the labeling was concentrated near the plasma membrane (Figures 5A,B), consistent with the known role of KCC2 in mediating chloride extrusion across the neuronal membrane. Importantly, KCC2 was also detected in a subset of presynaptic terminals, identified by the presence of synaptic vesicles ranging from 40 to 60 nm in diameter (Figures 5C,D). Although the density of labeling in presynaptic terminals was lower than in postsynaptic sites, gold particles were observed both near the membrane – suggesting potential involvement in chloride extrusion – and within the cytoplasm, possibly reflecting intracellular trafficking of KCC2.

While qualitative in nature, these findings provide supportive ultrastructural evidence for the presence of KCC2 in some presynaptic terminals within the MPN. This observation aligns with our electrophysiological results suggesting that spontaneous glutamate release may be modulated by KCC2 activity. Together, the ultrastructural and electrophysiological data are consistent with a presynaptic role for KCC2 in regulating glutamatergic synaptic transmission in the MPN.

## 4 Discussion

In the present study, we show that activation of chloride-permeable GABA_A_Rs and GlyRs expressed in the presynaptic terminals innervating neurons in the MPN modulates glutamate release. By using dissociated neurons with functionally preserved presynaptic terminals, we were able to selectively examine the local effects of activating presynaptic GABA_A_Rs and GlyRs while excluding contributions from somatic or proximal axonal compartments of the presynaptic neurons. Under these conditions, application of the neurosteroid allopregnanolone and the GABA_A_R agonist muscimol predominantly potentiated glutamate release, consistent with a depolarizing action at the presynaptic terminal mediated by chloride efflux resulting from an elevated intraterminal chloride concentration. In contrast, activation of presynaptic GlyRs by glycine application elicited a more heterogeneous response, causing both potentiation and, in some cases, a reduction of glutamate release. This variability strongly suggests terminal-specific differences in chloride regulation across presynaptic glutamatergic terminals in the MPN, as the inhibitory responses to glycine require a low intraterminal chloride concentration likely maintained by KCC2, which would allow chloride influx and hyperpolarization upon GlyR activation. The presence of KCC2 in a subset of presynaptic terminals is suggested by our immunogold staining results, which indicate KCC2 localization within presynaptic compartments in the MPN.

Potentiation of glutamate-mediated sEPSC frequency by allopregnanolone in a substantial proportion of the MPN cells tested, together with the sensitivity of this effect to picrotoxin, is consistent with the mechanism described in our previous report (Haage et al., 2002), where allopregnanolone-induced activation of presynaptic chloride permeability was shown to depolarize GABAergic terminals and thereby enhance GABA release onto MPN neurons. Similar presynaptic facilitation of glutamate release via GABA_A_R-dependent depolarization has been reported in the hippocampus (Stell, 2011; Iwata et al., 2013), whereas in cortical neurons allopregnanolone was shown to suppresses glutamate release, likely through inhibition or inactivation of presynaptic calcium channels (Long et al., 2009; Chang et al., 2019), underscoring region- and activity-dependent divergence in neurosteroid signaling. Interestingly, in the present study sIPSC responses did not always mirror sEPSC responses to allopregnanolone application in the same neurons, suggesting either differential expression or functional coupling of presynaptic chloride-permeable receptors at glutamatergic versus GABAergic terminals. Such synapse-specific modulation could represent a potential mechanism through which allopregnanolone might influence excitatory-inhibitory balance within the MPN. In turn, modulation of this balance may contribute to changes in MPN output under physiological conditions associated with fluctuating neurosteroid levels and, thus, affecting key instinctive behaviors governed by the MPN, including maternal care, sexual behavior, and social interaction (Láng et al., 2024).

Our further experiments with selective activation of presynaptic GABA_A_Rs provided additional support to the depolarization-induced potentiation of glutamate release. Specifically, application of the GABA_A_R-selective agonist muscimol, increased sEPSC frequency in the majority of tested MPN neurons. This facilitatory effect was abolished by the NKCC1 blocker bumetanide, indicating that GABA_A_R-mediated enhancement of glutamate release depends on NKCC1-driven elevation of intraterminal chloride concentration, leading to depolarizing chloride efflux upon receptor activation. Similar mechanisms were described in noradrenergic neurons of the locus coeruleus (Koga et al., 2005), neurons of the ventromedial hypothalamus (Jang et al., 2001) and in cerebellar Purkinje cells (de San Martin et al., 2017).

Importantly, the potentiating effect of presynaptic GABA_A_R activation was TTX-sensitive, consistent with observations in other brain regions, including cerebellar parallel fibers (Stell et al., 2007) and the hippocampus (Yamamoto et al., 2011; Kim et al., 2011). This suggests that GABA_A_R-induced depolarization is sufficient to reach the activation threshold of voltage-gated sodium channels but insufficient to directly activate presynaptic calcium channels. This is consistent with the predominance of high-voltage-activated calcium channels in MPN presynaptic terminals (Haage et al., 1998; Druzin et al., 2002), which open at more depolarized potentials than sodium channels (Sundgren-Andersson and Johansson, 1998; Simms and Zamponi, 2014; Ahern et al., 2016). Thus, sodium channel activation is likely required for presynaptic voltage-gated calcium channel opening, providing a plausible explanation for the TTX sensitivity of GABA_A_R-induced potentiation of sEPSC. It should be noted, however, that the absence of bicarbonate in our recording solutions may shift the membrane potential to more negative values (Kulik et al., 2000; Khazipov et al., 2004), which could partly contribute to the lack of direct activation of voltage-gated calcium channels. While this factor may affect the absolute chloride driving force, it does not alter the relative pharmacological effects observed here, which remain consistent with a chloride-dependent modulation of glutamate release.

In addition to GABA_A_Rs, allopregnanolone may modulate GlyRs, as previously demonstrated in cultured spinal neurons (Jiang et al., 2006) and in hippocampal and cerebellar Purkinje neurons (Bukanova et al., 2020). Such modulation may contribute to the allopregnanolone effects on glutamate release observed in the present study. Consistent with this, glycine produced bidirectional effects on sEPSC frequency in MPN neurons. In a large group of tested cells, glycine increased sEPSC frequency, resembling the facilitatory effects of muscimol and suggesting a depolarizing action of presynaptic GlyR activation. Similar depolarizing glycinergic effects have been reported in the brainstem, spinal cord, and midbrain (Turecek and Trussell, 2001; Jeong et al., 2003; Choi et al., 2013).

In contrast, glycine suppressed glutamate release in the remaining responsive MPN neurons. This dual action may explain why allopregnanolone-induced potentiation of glutamate release was observed in a smaller proportion of neurons compared with muscimol, as allopregnanolone may simultaneously activate presynaptic GABA_A_Rs that consistently enhance glutamate release and GlyRs that can either facilitate or inhibit release.

Presynaptic inhibition mediated by increased chloride conductance has previously been attributed to depolarization-dependent inactivation of voltage-gated sodium channels or to prevention of depolarization by shunting (Rudomin and Schmidt, 1999; Kullmann et al., 2005; Betelli et al., 2015). However, the TTX insensitivity of glycine-induced inhibition observed in our experiments is inconsistent with these mechanisms. Instead, our findings support a model in which glycine activates GlyRs under conditions of low intraterminal chloride concentration, leading to chloride influx and hyperpolarization of presynaptic terminals. This hyperpolarization reduces the probability of spontaneous activation of voltage-gated calcium channels and thereby suppresses glutamate release. Such a mechanism requires active chloride extrusion, likely mediated by KCC2 activity. Supporting this hypothesis, pharmacological blockade of KCC2 abolished glycine-induced inhibition. Although the blocker we used has recently been reported to also affect KCC1 (Zhao Y. et al., 2022), the expression of KCC1 in central neurons is relatively low (Kaila et al., 2014) and is therefore unlikely to significantly contribute to the effects we observed.

Further supporting a potential involvement of KCC2, we detected KCC2 immunolabeling in a subset of presynaptic terminals in the MPN. However, KCC2 labeling was not restricted to presynaptic sites and was also observed postsynaptically. This distribution is consistent with previous reports describing KCC2 localization in close proximity to glutamatergic terminals (Gulyás et al., 2001), although it has more commonly been reported in peri- and extrasynaptic regions (Barthó et al., 2004). Taken together, these observations suggest that KCC2 may also be present and functionally relevant in presynaptic terminals of central neurons.

Thus, the presence of functionally active KCC2 in presynaptic terminals may determine the effect of chloride-permeable receptor activation on neurotransmitter release. In terminals expressing KCC2, presynaptic receptor activation leads to chloride influx and membrane hyperpolarization, resulting in inhibition of neurotransmitter release. In contrast, in terminals lacking KCC2, the same receptors may exert depolarizing and excitatory effects. The outcome of receptor activation further depends on receptor subunit composition and activation mode, which can range from phasic activation (de San Martin et al., 2017) to tonic activation driven by neurotransmitter spillover, circulating neurosteroids, or spontaneous ligand-independent channel opening (Alle and Geiger, 2007; Ruiz et al., 2010).

The exact mechanisms responsible for presynaptic GABA_A_R and GlyR activation in the MPN remain largely unknown, but likely include allopregnanolone effects as the MPN is a well-established target of neurosteroid signaling (Henderson, 2007). Additionally, astrocytes may contribute to GABA_A_R and GlyR activation through the activity-dependent release of GABA, glycine and taurine (Lee M. et al., 2011; Choe et al., 2012; Bhattarai et al., 2015). In this context, presynaptic GABA_A_Rs and GlyRs may contribute to bidirectional glia–neuron signaling in the MPN, providing additional mechanisms of synaptic regulation. This idea is further supported by recent findings demonstrating that KCC2 can reverse its transport direction to clear extracellular K^+^ during periods of elevated glutamatergic activity, highlighting KCC2 as a potential regulator of glutamate synapses (Byvaltcev et al., 2023).

In summary, our findings suggest that presynaptic chloride homeostasis may influence how GABA_A_Rs and GlyRs regulate glutamate release in the MPN. The results are consistent with the presence of functionally active KCC2 in a subset of GlyR-expressing presynaptic terminals and raise the possibility that this transporter contributes to the ability of the same chloride-permeable receptors to exert either facilitatory or inhibitory effects, depending on terminal-specific chloride regulation. Such synapse-specific regulation could represent a mechanism for dynamically adjusting excitatory–inhibitory balance within MPN circuits. This mechanism may be particularly relevant during behavioral states associated with fluctuations in neurosteroid levels, including reproduction, parental behavior, and social interaction.

## Data Availability

The raw data supporting the conclusions of this article will be made available by the authors, without undue reservation.
